# Exploring telomere length in mother–newborn pairs in relation to exposure to multiple toxic metals and potential modifying effects by nutritional factors

**DOI:** 10.1186/s12916-019-1309-6

**Published:** 2019-04-11

**Authors:** Maria Herlin, Karin Broberg, Annachiara Malin Igra, Huiqi Li, Florencia Harari, Marie Vahter

**Affiliations:** 1 Institute of Environmental Medicine, Karolinska Institutet, P.O. Box 210, SE-171 77 Stockholm, Sweden; 2 Division of Occupational and Environmental Medicine, Department of Laboratory Medicine, Lund University, Lund, Sweden; 3 Department of Occupational and Environmental Medicine, Sahlgrenska University Hospital and University of Gothenburg, Gothenburg, Sweden; 4 Current Address: Department of Environmental Research and Monitoring, Swedish Museum of Natural History, Stockholm, Sweden; 5 Current Address: Department of Occupational and Environmental Medicine, Sahlgrenska University Hospital and University of Gothenburg, Gothenburg, Sweden

**Keywords:** Early life programming, Arsenic, Antimony, Boron, Lead, Lithium, Zinc, Nutrients, Telomeres

## Abstract

**Background:**

The uterine environment may influence telomere length at birth, which is essential for cellular function, aging, and disease susceptibility over the lifespan. However, little is known about the impact of toxic chemicals on early-life telomeres. Therefore, we assessed the potential impact of multiple toxic metals on relative telomere length (rTL) in the maternal blood, cord blood, and placenta, as well as the potential modifying effects of pro-oxidants.

**Method:**

In a mother–child cohort in northern Argentina (*n* = 169), we measured multiple toxic metals in the maternal blood or urine collected during late pregnancy, as well as the placenta and cord blood collected at delivery, using inductively coupled plasma mass spectrometry (ICP-MS). We assessed associations of log_2_-transformed metal concentrations with rTL, measured in maternal and cord blood leukocytes and the placenta by real-time PCR, using multivariable-adjusted linear regression. Additionally, we tested for modifications by antioxidants (zinc, selenium, folate, and vitamin D_3_).

**Results:**

Exposure to boron and antimony during pregnancy was associated with shorter maternal rTL, and lithium with longer maternal rTL; a doubling of exposure was associated with changes corresponding to 0.2–0.4 standard deviations (SD) of the rTL. Arsenic concentrations in the placenta (*n* = 98), blood, and urine were positively associated with placental rTL, about 0.2 SD by doubled arsenic. In the cord blood (*n* = 88), only lead was associated with rTL (inversely), particularly in boys (*p* for interaction 0.09). Stratifying by newborn sex showed ten times stronger association in boys (about 0.6 SD) than in girls. The studied antioxidants did not modify the associations, except that with antimony.

**Conclusions:**

Elevated exposure to boron, lithium, arsenic, and antimony was associated with maternal or newborn rTL in a tissue-specific, for lead also sex-specific, manner. Nutritional antioxidants did not generally influence the associations.

**Electronic supplementary material:**

The online version of this article (10.1186/s12916-019-1309-6) contains supplementary material, which is available to authorized users.

## Background

Telomeres are nucleotide sequences at the end of chromosomes of importance for maintaining genomic integrity [1, 2]. They are shortened by each cell division as a normal cellular process, eventually reaching a critically short length of the chromosomes that activates the replicative senescence and aging of tissues and organs and, in turn, increases the risk of diseases [3]. The activity of telomerase, the main telomere-maintaining enzyme, is strictly regulated in both the placenta and embryo and has been suggested to be an important factor for a successful fetal development via fetal programming [4]. Still, the telomeres of the developing organism exhibit plasticity [5]. Thus, the uterine environment may influence fetal telomere length (TL). In particular, associations with nutritional status, especially of zinc, folate, and vitamin D, have been reported [6–9]. There is emerging evidence that also factors, such as maternal obesity [10], stress [11, 12], and smoking [13, 14], may influence child TL. This may have long-term effects, as TL at birth is believed to contribute substantially to the TL throughout life [5, 15, 16].

Data on the impact of toxic chemicals on early-life TL is more limited. A few studies concerned air pollution [17–19] and persistent organic pollutants [20]. Also, certain toxic metals, especially those causing oxidative stress, have been found to be associated with telomere length. Telomeres are susceptible to oxidative stress due to their high content of guanines and their inefficient repair system of single-strand breaks [21, 22]. Thus, cadmium exposure during pregnancy has been reported to be inversely associated with placental TL [23] and cord blood TL [24, 25], while concurrent exposure was inversely associated with salivary TL in adolescents [26]. Also, lead exposure was inversely associated with blood TL in children aged 6–10 years [27]. Longer TL has been associated with exposure to arsenic, another pro-oxidant, but the association seems to be dose-dependent. Low-level childhood arsenic exposure was found to be positively associated with blood TL in 9-year-old children, while the association turned inverse at urinary arsenic concentrations exceeding 45 μg/L [28]. In support, in vitro studies using the human cord blood showed that very low concentrations of arsenic (sub-nM) increased telomerase mRNA and protein expression, while 1 μM arsenic decreased telomerase expression and TL [29]. Taken together, these findings indicate that early-life telomeres may be susceptible to toxic metals. Therefore, the aim of the present study was to assess the potential impact of environmental exposure to multiple toxic metals on TL in the placenta and cord blood, and whether any association could be modified by antioxidants. For comparison, we also evaluated associations with TL in the maternal blood during pregnancy.

## Methods

### Study population

The study was based on a mother–child cohort in the Andean part of the Salta province, northern Argentina, designed to evaluate potential health effects in relation to early-life exposure to multiple toxic metals in drinking water and food [30–32]. It is known since long that elevated concentrations of arsenic, a potent toxicant and carcinogen, are common in the drinking water in this area [33]. More recently, it was discovered that the water in some of the villages also contained elevated concentrations of lithium, boron, and cesium [34]. The study area included the main village San Antonio de los Cobres, with about 5900 inhabitants, and nine smaller villages, located at a distance of 40–216 km (altitude of 3180–4070 m a.s.l.). The antenatal and infant care was organized through the hospital in San Antonio de los Cobres. All pregnant women in this area with estimated delivery date between October 2012 and December 2013 were invited to participate in the study. Out of a total of 221 pregnant women, 194 became enrolled. More details about the study area, recruitment of the cohort, and loss to follow-up have been described elsewhere [31].

### Data and sample collection

Details concerning investigations and sampling have been described elsewhere [31]. In brief, at the first meeting, the pregnant women were interviewed about age, last menstrual period (LMP), pre-pregnancy weight, parity, family income, education level, smoking, alcohol consumption, coca chewing, and personal and family history of diseases. At each visit, the women were asked about potentially encountered health problems during pregnancy, and blood and urine samples were collected for assessment of exposure to toxic and essential elements. Weight and lean body mass were measured, as well as height at the first visit [31].

Maternal venous blood samples were collected in Trace Elements Sodium Heparin tubes and in Trace Elements Serum Clot Activator tubes (Vacuette®; Greiner Bio-One International GmbH, Kremsmünster, Austria). Serum and plasma were fractionated by centrifugation (3000 rpm, 10 min) 15 min after blood withdrawal. Blood for DNA extraction was collected in EDTA tubes (Vacuette® K3EDTA; Greiner Bio-One International GmbH). Because of the large study area with many women living far from the village primary health care clinic, it was not possible to obtain fasting blood samples or to collect samples at a fixed time of the day. Midstream spot urine samples were collected in disposable trace element-free plastic cups and immediately transferred to 20-mL polyethylene bottles (Zinsser Analytic GMBH, Frankfurt, Germany), shown to be essentially free of trace element contamination.

Cord blood samples and the whole placenta were collected at delivery and frozen at − 20 °C. The blood was collected in Trace Elements Sodium Heparin tubes and in Trace Elements Serum Clot Activator tubes (Vacuettes; Greiner Bio-One International GmbH) and processed in the same way as the maternal blood samples. All specimens were transported on ice to Karolinska Institutet, Stockholm, for analysis of trace elements. After thawing of the placenta samples, the amnion was removed. Thereafter, three biopsies (subsamples of both maternal and the fetal tissues across the placenta) of ∼ 9 cm^3^ were cut out at different distances from the cord in each placenta, using a ceramic knife tested free for trace element contamination. Each piece was transferred to a 20-mL polyethylene tube (Zinsser Analytic GMBH, Frankfurt, Germany) and frozen at − 80 °C until analyses. In addition, one biopsy of ∼ 5 cm^3^ was cut out about 5 cm from the umbilical cord insertion and 1.0–1.5 cm below the fetal membranes, in a way to obtain homogenous samples from the placental villous parenchyma. Approximately 25 mg of the latter biopsy was used to extract DNA, after as much as possible of the maternal blood was removed by washing with sterile phosphate-buffered saline. Previous studies found no significant influence of sampling site on placental TL [35].

Birth weight was measured using Seca 725 Mechanical Beam Baby Scale (Brooklyn, NY, USA), birth length using a portable wood infantometer to the nearest 5 mm with the child in supine position, and head circumference using a soft, non-stretchable plastic tape line. Gestational age at birth was calculated by subtracting the date for LMP from the date of birth. In the few cases of missing LMP, the ultrasound estimation was used.

### Measurement of telomere length

The relative telomere length (rTL) was measured in maternal blood leukocytes (blood samples collected in late pregnancy, mainly third trimester), cord blood leukocytes, and placental tissue. We obtained high-quality DNA and measured rTL in 169 blood samples of the pregnant women, 99 of their placentas, and 98 cord blood samples of their babies. DNA from the maternal whole blood was extracted using QIAamp DNA blood Mini kit (Qiagen, Hilden, Germany), from the cord blood using E.Z.N.A. Blood DNA Mini kit (OMEGA Bio-Tek, Inc., USA), and from the placenta using QIAamp DNA Mini kit (Qiagen). The rTL was measured as the ratio between the signal intensity of the telomere sequences and the signal intensity of a single-copy gene (hemoglobin β chain, *HBB*), using a real-time PCR (7900HT, Applied Biosystems, Foster City, CA, USA) as described previously [36]. Primers for telomere and *HBB* amplicons as well as the thermal cycling profiles are presented in Additional file 1: Table S1. In short, PCR reactions for telomere and *HBB* were run on separate plates with respective settings. The master mix for telomere was prepared with telomere-specific primers (0.45 μM of each primer), PCR buffer (1×) (all PCR reagents from Life Technologies, Carlsbad, CA, USA), 1.75 mM MgCl_2_, 0.8 mM dNTPs, 0.3 mM SYBR Green, Rox (1×), and 0.5 U Platinum *Taq* polymerase. Master mix for *HBB* was prepared with *HBB* primers (0.40 μM for each primer) and KAPA SYBR FAST qPCR Kit Master Mix (1×) ABI Prism (Kapa Biosystems, Woburn, MA, USA). One calibrator DNA was prepared by pooling DNA from samples randomly selected. The calibrator DNA was then serially diluted to produce concentrations of 0.31–20 ng/μL for the standard curve. The standard curve and a negative control were included in each run. All samples, DNA standards, and negative controls were run in triplicate. *R*^2^ for each standard curve was > 0.99. If the standard deviations of cycle threshold of the triplicate were above 0.1, the deviated one would be excluded. The relative length of the telomeres was obtained through calculating the ratio of the telomere repeat product to the single-copy gene product (here *HBB*) for each individual. The telomere length ratio is an arbitrary value. The coefficient of variation for the telomere measurement was 8.0% based on 11 repeats of the reference sample.

### Measurement of toxic metals and micronutrients

Concentrations of the toxic elements lithium, boron, arsenic, cadmium, antimony, cesium, and lead were measured in the maternal whole blood, serum or urine (depending on their suitability as exposure biomarker [31, 32] and considering potential collinearity), placenta, and umbilical cord blood. We also measured essential trace elements with anti-oxidative properties, i.e., zinc and selenium [8]. All element concentrations were determined using inductively coupled plasma mass spectrometry (ICP-MS; Agilent 7700x, Agilent Technologies, Tokyo, Japan), with the collision/reaction cell in no gas, hydrogen, or helium mode. Before the ICP-MS analysis, urine samples were diluted 1:10 with 1% nitric acid (65% *w*/*w*, ppb-trace analysis grade, Scharlau, Scharlab S.L., Sentmenat, Spain) [34]. Aliquots (0.2 mL) of whole blood or serum samples were diluted 1:25 with an alkali solution consisting of 1-butanol 2% (*w*/*v*), EDTA 0.05% (*w*/*v*), triton X-100 0.05% (*w*/*v*), NH_4_OH 1% (*w*/*v*), and internal standards (20 μg/L) [37]. The mixture was sonicated for 5 min and centrifuged at 2000 rpm for 5 min (MSE centrifuge, Super Minor, MSE (UK) Ltd., London, England) before ICP-MS analysis. This method was found to provide more reliable results for blood lithium and boron than acid digestion [37].

After thawing, about 1 g of each placenta sample was mixed with 2 mL of nitric acid (65% *w*/*w*, ppb-trace analysis grade, Scharlab S.L.) and 3 mL of deionized water and thereafter digested at 250 °C for 30 min in a Milestone ultraCLAVE II microwave digestion system (EMLS, Leutkirch, Germany). After reaching room temperature, the samples were transferred to acid-washed polyethylene tubes and diluted with deionized water until 10.8 g of weight, to get a 20% nitric acid concentration. This solution was then analyzed for multiple elements by the ICP-MS instrument mentioned above. For ten of the placentas, we measured multiple metals in all three specimens and found small differences, but the one closest to the cord (about 5 cm) appeared most representative of the whole placenta (Spearman’s correlation *r*_S_ > 0.73 for all elements, except for antimony which had 0.66). Also, it was closest to the biopsy used for DNA extraction.

Results below the limit of detection (LOD), calculated as three times the blank values, were used at face values. Negative values, mainly for cadmium in the cord blood and antimony in the placenta, were recalculated to LOD divided by the square root of 2. The concentrations in urine were corrected for variation in urine dilution by adjustment to the average osmolality (694 mOsm/kg), measured by a digital cryoscopic osmometer (Osmomat®030, Gonotec Gesellschaft für Meβ- und Regeltechnik mbH, Berlin, Germany).

The concentrations of serum folate and plasma vitamin D (25-hydroxivitamin D_3_), which also have anti-oxidative properties and have been linked to TL [6, 7, 9], were measured at Clinical Chemistry, Lund University Hospital, Sweden, using standard clinical analytical methods.

### Statistical methods

Statistical analyses were performed using Stata 11.2 (StataCorp LP, TX, USA). Bivariate associations between telomere length and concentrations of trace elements and other nutritional factors in blood, plasma/serum, urine, and placenta were initially assessed with Spearman’s rank correlation test (*r*_S_) and visual evaluation of scatter plots of the outcomes against the different exposures.

Multivariable-adjusted linear regression analysis was then performed to evaluate the associations between exposures and rTL in the different tissues. We used the false discovery rate (FDR) of multiple testing correction according to the Benjamini–Hochberg FDR method [38] and applied a significance threshold of *q* < 0.05. Basically, the obtained lowest *p* value was multiplied by the number of tests performed for each media (maternal blood, placenta, and umbilical cord blood), the next lowest by the number of analyses minus one, and so on. The same process was applied for the evaluation of the micronutrients evaluated as potential modifiers. As the concentrations of most toxic elements were not normally distributed, they were logarithm-transformed (log_2_). As such, they met the assumption of homogenous variances and provided the best fit of the data. We chose log_2_ transfomation of the metal concentrations because it provides a simple interpretation of the *B*-coefficient in the linear regression analysis, i.e., the mean change in outcome associated with doubling of the concentration. The crude model (model 1) included the exposure only, while the adjusted model (model 2) included maternal age, education (years at school), and pre-pregnancy body mass index (BMI; weight/height^2^) as covariates. Both age and pre-pregnancy BMI have been found to be associated with shorter telomeres [3, 10]. Maternal education is the main socioeconomic factor available. When evaluating rTL in the placenta and cord blood, we additionally adjusted for gestational week at birth, and for rTL in the cord blood also birth weight, as those have been found to be associated with cord blood rTL [39]. We additionally adjusted for other toxic metals as appropriate based on previous knowledge and their current associations with rTL. As lithium, boron, and arsenic often occurred at elevated levels in the same drinking water source, the exposure markers were correlated. In order to decrease collinearity, we used serum boron, whole blood lithium, and urinary arsenic (*r*_S_ for serum boron and blood lithium 0.77, for serum boron and urinary arsenic 0.39, and for blood lithium and urinary arsenic 0.53) in the evaluation of association with maternal rTL. For the associations with placenta and cord blood rTL, we used concentrations of all metals in respective tissue. We tested for sex differences by including a multiplicative interaction term. If significant, we stratified the model by sex and compared the estimates for boys and girls (Wald test).

We selected nutritional factors with antioxidant properties (zinc, selenium, folate, and vitamin D_3_) that might act as modifier of the associations between toxic metals and rTL. First, we evaluated the associations between these nutrients and rTL in the different tissues. In the models with vitamin D_3_, we additionally adjusted for the season of sampling (summer/fall/spring/winter).

In sensitivity analyses, we tested adjusting the cord blood models also for maternal rTL, as that could mediate associations observed with cord blood rTL. Also, we evaluated additional adjustment with age of the fathers, which previously has been found to be associated with longer telomeres in the offspring [15, 40]. In additional sensitivity analyses, we excluded a few extreme values of some element concentrations in order to test if associations were driven by such values.

## Results

### General characteristics and exposure

General characteristics of the cohort are presented in Table 1 by tertiles of rTL in the maternal blood and umbilical cord blood. Relative TL was longer in the cord blood (1.26 ± 0.12) compared with the placenta (0.77 ± 0.21) and the maternal blood in late pregnancy (0.99 ± 0.31), and the inter-individual variation in rTL was markedly smaller in the cord blood than in the maternal blood. Cord blood rTL correlated moderately with rTL in the placenta (*r*_S_ = 0.37, *p* < 0.001) and weakly with the maternal blood (*r*_S_ = 0.20, *p* = 0.08), while rTL in the maternal blood and placenta were not correlated (*r*_S_ = − 0.10, *p* = 0.38). Neither cord blood rTL nor placental rTL differed by newborn sex. Maternal rTL decreased with age (*r*_S_ = − 0.20, *p* = 0.009; about 1% a year). Other factors which appeared to vary across the tertiles of maternal rTL (Table 1) were serum concentrations of folate (*r*_S_ = − 0.21; *p* = 0.009), but not Hb, parity, pre-pregnancy weight, or BMI. The rTL of the mothers chewing coca leaves did not differ from those not using coca (*r*_S_ = 0.005; *p* = 0.95). Only one woman reported smoking during pregnancy, and five women reported that they used alcohol, however, very seldom. Paternal age was not correlated with cord blood rTL (*r*_S_ = 0.08; *p* = 0.46), but weakly correlated with placental rTL (*r*_S_ = 0.22; *p* = 0.046).

**Table 1:** General characteristics of the studied mothers and newborns by tertiles of relative telomere length (rTL) in maternal blood and umbilical cord blood, respectively

	Tertile 1	Tertile 2	Tertile 3	*p* ^a^
Mothers	*n* = 57	*n* = 56	*n* = 56	
Leukocyte rTL	0.67 (0.33–0.83)	0.98 (0.84–1.1)	1.3 (1.1–1.9)	< 0.001
Maternal age (years)	26 (15–41)	24 (12–40)	22 (14–40)	0.20
Maternal education (years)	9 (0–16)	10 (0–14)	9 (0–17)	0.36
Parity (*n*)	1 (0–11)	1 (0–12)	1 (0–7)	0.48
Pre-pregnancy weight (kg)	52 (38–86)	52 (39–75)	52 (40–74)	0.67
Height (cm)	153 (134–166)	152.5 (144–168)	152 (140.5–163)	0.85
Serum folate (nmol/L)	27 (11.3–45.4)	25.9 (14.2–36.9)	24.7 (15.7–34.9)	0.04
Serum vitamin D_3_ (nmol/L)	43 (13–88)	41 (20–85)	44 (18–82)	0.40
Hb (g/L)	137 (96–186)	137 (95–158)	138 (117–166)	0.62
Coca chewing	21 (37%)	30 (55%)	24 (43%)	0.26
Placental rTL (*n* = 98)	0.73 (0.53–1.2)	0.79 (0.37–1.2)	0.74 (0.33–1.1)	0.79
Cord blood rTL (*n* = 98)	1.2 (1.0–1.5)	1.3 (0.98–1.4)	1.3 (1.1–1.6)	0.15
Newborns	*n* = 33	*n* = 33	*n* = 32	
Cord blood rTL	1.2 (0.98–1.2)	1.3 (1.2–1.3)	1.4 (1.3–1.7)	< 0.001
Sex (boys; *n* (%))	10 (31%)	16 (48%)	15 (47%)	0.42
Gestational age at birth (weeks)	39 (32–42)	39 (34–43)	39 (34–42)	0.97
Birth weight (g)	3085 (1820–4500)	3050 (2400–3730)	3000 (2560–3650)	0.52
Birth length (cm)	49 (43–51)	48 (45–51)	48 (41–51)	0.41
Head circumference (cm)	34 (31–40)	33 (30–36)	34 (32–36)	0.35

Values given are median concentration (range)

^a^*p* value for difference across telomere tertiles (Kruskal–Wallis test)

Element concentrations in the various sample types are presented in Table 2. Particularly wide variations were found for lithium, boron, arsenic, and cesium. There was a strong correlation (*r*_S_ = 0.89) between blood arsenic and urinary arsenic (median 124, range 13–2450 μg/L, osmolality adjusted), and also between blood and placental arsenic (*r*_S_ = 0.68). From a toxicological point of view, some women also had elevated blood lead concentrations (range 6.9–99 μg/L). Cadmium showed the most pronounced accumulation in the placenta, 22 times the concentration in the maternal blood, which, however, was quite low (0.04–0.47 μg/L). This resulted in very low concentrations in the cord blood, below the detection limit of 0.01 μg/L for a majority of the newborns. Most other metals appeared to pass to the fetus.

**Table 2:** Concentrations of toxic and essential elements in the maternal whole blood or serum (average concentrations across the second and third trimesters) and in the placenta and umbilical cord blood or serum

	Maternal blood/serum (*n* = 169)	Placenta (*n* = 99)	Cord blood/serum (*n* = 98)
Lithium (μg/L^a^ or μg/kg^c^)	24 (1.9–145)	38 (5.2–143)	48 (9.5–156)
Boron (μg/L^b^ or μg/kg^c^)	135 (0.13–605)	143 (1.1–605)	193 (69–698)
Arsenic (μg/L^a^ or μg/kg^c^)	2.2 (0.42–23)	8.4 (1.6–51)	2.3 (0.47–14)
Cadmium (μg/L^a^ or μg/kg^c^)	0.16 (0.04–0.47)	3.6 (1.4–11)	<LOD (<LOD–0.12)^d^
Antimony (μg/L^a^ or μg/kg^c^)	2.6 (1.2–46)	0.024 (<LOD–1.4)^e^	2.5 (0.84–18)
Cesium (μg/L^a^ or μg/kg^c^)	110 (2.5–685)	419 (14–1526)	153 (7.2–690)
Lead (μg/L^a^ or μg/kg^c^)	21 (6.9–99)	5.8 (1.2–38)	14 (6.3–60)
Zinc (mg/L^a^ or mg/kg^c^)	6.1 (4.4–14)	8.6 (6.7–13)	1.8 (1.2–4.8)
Selenium (μg/L^b^ or μg/kg^c^)	86 (50–136)	176 (120–304)	49 (34–105)

Values given represent median concentration (range)

^a^Unit of concentrations in whole blood

^b^Unit of concentrations in serum

^c^Unit of concentrations in the placenta

^d^71% below LOD (0.02 μg/kg)

^e^45% below LOD (0.01 μg/L)

### Associations of biomarkers of toxic metals with rTL in the maternal blood

Scatter plots of the correlations between the toxic metals and rTL in the maternal blood are shown in Additional file 1: Figure S1. Unadjusted (model 1) and adjusted (models 2–4) associations between the toxic metals (log_2_-transformed average concentrations across the second and third trimesters) and rTL in the maternal blood are shown in Table 3. In the multivariable-adjusted regression models (model 2, adjusted for maternal age, pre-pregnancy BMI, and years of education), maternal blood lithium was positively associated with rTL, while serum boron and blood antimony were inversely associated with rTL. Including multiple toxic metals in the same model (model 3) increased the estimates for lithium and boron markedly (BH-adjusted *q* values 0.012 and 0.007, respectively), but decreased slightly the estimate for antimony (BH-adjusted *q* value 0.025). Analysis of collinearity by the variance inflation factor showed 1.1–2.6, indicating an acceptable linear combination of variables. Excluding four high log_2_ blood antimony concentrations (Additional file 1: Figure S1; corresponding to blood antimony > 20 μg/L, all others being < 8 μg/L) strengthened the association by 35% (*B* = 0.123; 95% CI − 0.214, − 0.032; *p* = 0.008; BH-adjusted *q* value 0.040). There were no interactions with sex (*p* for interaction was 0.873 for lithium, 0.482 for boron, and 0.866 for antimony).

**Table 3:** Multivariable-adjusted linear regression between concentrations of toxic metals (log_2_-transformed) in the maternal blood/serum, placenta, or cord blood/serum and relative telomere length in the maternal blood, placenta, or cord blood

Exposure biomarker	Model	Maternal blood rTL (*n* = 169)		Placenta rTL (*n* = 98)		Cord blood rTL (*n* = 88)	
		*B* (95% CI)	*p* value	*B* (95% CI)	*p* value	*B* (95% CI)	*p* value
Lithium		Blood (μg/L)		Placenta (μg/kg)		Cord blood (μg/L)	
	1	0.031 (− 0.015, 0.076)	0.188	0.033 (− 0.013, 0.079)	0.154	0.022 (− 0.007, 0.051)	0.132
	2	0.030 (− 0.016, 0.076)	0.195	0.030 (− 0.016, 0.076)	0.202	0.011 (− 0.020, 0.042)	0.478
	3	0.107 (0.039, 0.176)	0.002				
	4	0.104 (0.035, 0.172)	0.003				
Boron		Serum (μg/L)		Placenta (μg/kg)		Cord serum (μg/L)	
	1	− 0.035 (− 0.001, 0.00007)	0.041	0.013 (− 0.24, 0.051)^c^	0.488	0.051 (0.013, 0.090)	0.009
	2	− 0.034 (− 0.068, − 0.001)^a^	0.045	0.009 (− 0.028, 0.046)^c^	0.630	0.030 (− 0.012, 0.071)	0.158
	3	− 0.075 (− 0.119, − 0.031)^a^	0.001				
	4	− 0.072 (− 0.115, − 0.028)^a^	0.001				
Arsenic		Urine (μg/L)		Placenta (μg/kg)		Cord blood (μg/L)	
	1	0.011 (− 0.031, 0.053)	0.596	0.046 (− 0.005, 0.087)	0.028	0.012 (− 0.017, 0.040)	0.407
	2	0.014 (− 0.013, 0.089)	0.510	0.039 (− 0.002, 0.080)	0.060	− 0.002 (− 0.026, 0.030)	0.879
	3			0.051 (− 0.016, 0.119)	0.133		
	4			0.053 (0.012, 0.093)	0.011		
Cadmium		Blood (μg/L)		Placenta (μg/kg)		Maternal blood (μg/L)	
	1	− 0.069 (−1.43, 0.005)	0.068	− 0.012 (− 0.081, 0.057)	0.735	0.019 (− 0.010, 0.047)	0.197
	2	− 0.025 (− 0.107, 0.057)	0.545	− 0.047 (− 0.118, 0.025)	0.197	0.031 (− 0.013, 0.074)	0.162
	3			− 0.049 (− 0.120, 0.021)	0.167		
	4			− 0.028 (− 0.101, 0.045)	0.448		
Antimony		Blood (μg/L)		Placenta (μg/kg)		Cord blood (μg/L)	
	1	− 0.099 (− 0.160, − 0.038)	0.002	− 0.0002 (− 0.024, 0.025)	0.988	− 0.014 (− 0.046, 0.018)	0.377
	2	− 0.110 (− 0.171, − 0.049)^b^	< 0.001	− 0.003 (− 0.028, 0.023)	0.834	− 0.005 (− 0.036, 0.027)	0.774
	3	− 0.091 (− 0.154, − 0.028)^b^	0.005				
	4	− 0.065 (− 0.129, 0.001)^b^	0.045				
Cesium		Blood (μg/L)		Placenta (μg/kg)		Cord blood (μg/L)	
	1	0.012 (− 0.019, 0.044)	0.441	0.027 (− 0.011, 0.064)	0.162	− 0.012 (− 0.032, 0.008)	0.243
	2	− 0.0005 (− 0.032, 0.033)	0.975	0.018 (− 0.019, 0.055)	0.344	− 0.010 (− 0.031, 0.012)	0.385
Lead		Blood (μg/L)		Placenta (μg/kg)		Cord blood (μg/L)	
	1	0.037 (− 0.029, 0.104)	0.269	− 0.042 (− 0.085, 0.001)	0.057	− 0.046 (− 0.081, − 0.010)	0.013
	2	0.026 (− 0.043, 0.095)	0.458	− 0.029 (− 0.074, 0.016)	0.207	− 0.038 (− 0.074, − 0.002)	0.037
	3					− 0.040 (− 0.078, − 0.003)	0.040
	4					− 0.043 (− 0.079, − 0.006)	0.022

Model 1: Unadjusted

Model 2: Adjusted for maternal age, pre-pregnancy BMI, and education; placental rTL also for gestational age at birth; cord blood rTL also for gestational age at birth and birth weight, but not education

Model 3: Further adjusted for other metals: maternal rTL: blood lithium, serum boron, urinary arsenic, and blood antimony (if not dependent variable); placental rTL: all placenta metals; cord blood rTL: all cord blood metals

Model 4: Adjusted for nutrients. Maternal rTL: Model 3 was further adjusted for blood zinc and serum folate. Placental rTL: Model 2 was adjusted for placental Zn and vitamin D_3_. Cord blood rTL: Model 2 was further adjusted for cord blood zinc

^a^Excluding two outliers of serum boron < 1 log_2_ μg/L gave model 2: *B* = − 0.044; 95% CI − 0.097, 0.008; *p* = 0.097; model 3: *B* = − 0.117; 95% CI − 0.187, − 0.046; *p* = 0.001; and model 4: *B* = − 119; 95% CI − 0.189, − 0.049; *p* = 0.001

^b^Excluding two outliers of blood antimony > 4 log_2_ μg/L gave model 2: *B* = − 0.142; 95% CI − 0.232, − 0.052; *p* = 0.002; model 3: *B* = − 0.123; 95% CI − 0.214, − 0.032; *p* = 0.008; and model 4: *B* = − 0.090; 95% CI − 0.184, 0.004; *p* = 0.061

^c^Excluding one outlier of placenta boron < 4 log_2_ μg/kg gave model 1: *B* = − 0.028; 95% CI − 0.021, 0.076; *p* = 0.259; model 2: *B* = 0.021; 95% CI − 0.029, 0.070; *p* = 0.413

We found no associations of rTL in relation to concentrations of cadmium, cesium, or lead in the maternal whole blood, or with arsenic in urine (Table 3). Maternal urinary cadmium (median 0.10, range 0.04–2.1 μg/L), which reflects more long-term exposure than blood cadmium, gave similar association as blood cadmium (*B* = − 0.026; 95% CI − 0.085, 0.032; *p* = 0.375; model 2 with log_2_-transformed urinary cadmium).

When assessing associations between nutritional factors and maternal rTL (Additional file 1: Table S2), blood concentration of zinc was found to be positively associated with rTL (BH-adjusted *q* value 0.016 in model 2), while serum folate showed inverse associations with rTL (BH-adjusted *q* value 0.021). Entering these micronutrients in the same model did not markedly change the estimates (model 3, Additional file 1: Table S2). Adding serum zinc and folate to the models for the toxic metals decreased the estimate for antimony, but did not change those for lithium and boron (model 4, Table 3).

### Associations of biomarkers of toxic metals with rTL in the placenta

Scatter plots of the correlations between the toxic metals and rTL in the placenta are shown in Additional file 1: Figure S2. In the regression analysis, arsenic concentrations (log_2_-transformed) in the placenta were positively associated with placental rTL (Table 3, Additional file 1: Figure S2), and arsenic in blood and urine during pregnancy showed similar associations with placental rTL (blood: *B* = 0.051; 95% CI 0.005, 0.096; *p* = 0.029; urine: *B* = 0.036; 95% CI − 0.003, 0.076; *p* = 0.071; model 2). Further adjusting for other toxic elements in the placenta (lithium, boron, cadmium, antimony, cesium, and lead) increased the estimate for placental arsenic by about 30% (model 3, Table 3). However, testing for collinearity showed high variance inflation factors (1.1–6.0). Therefore, we also adjusted for maternal serum boron and blood lithium and cesium during pregnancy, instead of the concentrations in the placenta, and that decreased the variance inflation factor range to 1.1–2.5, but did not alter the overall estimate for placental rTL in relation to arsenic much (*B* = 0.043; 95% CI − 0.001, 0.086; *p* = 0.055).

Adjusting placental arsenic model 2 for placental zinc and plasma vitamin D_3_, which we found associated with placental rTL (Additional file 1: Table S2, BH-adjusted *q* values 0.162 and 0.048, respectively), did not markedly change the estimate (model 4, Table 3). However, it lowered the *p* value for placenta arsenic (BH-adjusted *q* value 0.056). The limited number of placentas did not allow for inclusion of both toxic metals and placental zinc and vitamin D_3_ in the same model. There was no interaction with sex (*p* for interaction was 0.865, model 4).

In sensitivity analysis, we tested if including paternal age (26.9 ± 7.3 years), instead of maternal age, changed the association of arsenic with placental rTL. Paternal age had little influence on the association (model 2: *B* = 0.041; 95% CI 0.001, 0.081; *p* = 0.045). However, paternal age as such was significant in the model (*B* = 0.008; 95% CI 0.002, 0.014; *p* = 0.006), slightly stronger than maternal age (model 2: *B* = 0.005; 95% CI − 0.002, 0.012; *p* = 0.131). The placental arsenic model 3 with paternal age explained 41% of the variation in placental rTL, compared to 32% in the model with maternal age.

We found no significant associations between the other toxic metals and placental rTL. As cadmium accumulates in the placenta (Table 2), we further adjusted the placental cadmium model 2 for placental arsenic (model 3, Table 3), which did not change the estimate, or for placental zinc and vitamin D_3_, which markedly decreased the estimate (model 4).

### Associations of biomarkers of toxic metals and nutritional factors with rTL in the cord blood

Scatter plots of the correlations between the toxic metals and rTL in the cord blood are shown in Additional file 1: Figure S3. In the unadjusted regression analysis, the boron concentrations in cord serum (log_2_-transformed) were positively associated with cord blood rTL (model 1, Table 3), but the estimate decreased after adjusting for mothers’ age, pre-pregnancy BMI, education, gestational age at birth, and birth weight (model 2). We found no significant association with cord blood rTL for lithium, arsenic, antimony, or cesium in the cord blood. Because the concentration of cadmium was below LOD in 71% of the cord blood samples, we used the maternal blood cadmium concentrations in the models, but it was not significant (Table 3).

The concentration of lead in the cord blood, on the other hand, was consistently inversely associated with cord blood rTL (models 1 and 2, Table 3; Additional file 1: Figure S3). The BH-adjusted *q* value was 0.091 and 0.259 for models 1 and 2, respectively. Further adjusting for other toxic metals (model 3) changed the estimate very little. Neither addition of cord blood zinc to model 2 changed the estimate. Of the nutritional factors, only cord blood concentration of zinc (median 1.9 mg/L) was found to be associated (inversely) with rTL in the cord blood (Additional file 1: Table S2).

Adding an interaction factor for sex and cord blood lead in model 2 showed *p* = 0.091, and stratifying the model by newborn sex (49 boys and 39 girls) showed about one hundred times stronger association in boys (model 2: *B* = − 0.064; 95% CI − 0.113, − 0.015; *p* = 0.012; *R*^2^ = 0.26) than in girls (*B* = − 0.0006; 95% CI − 0.054, 0.053; *p* = 0.983; *R*^2^ = 0.05). The difference was statistically significant (*p* = 0.015, Wald test). Scatter plots for cord blood rTL in relation to cord blood lead by sex are shown in Additional file 1: Figure S3.

In an additional sensitivity analysis, we found that adjusting for maternal leukocyte rTL only slightly decreased the estimates for lead (model 2, all newborns: *B* = − 0.034; 95% CI − 0.072, 0.003; *p* = 0.070), suggesting that the association was not mediated through the maternal rTL.

## Discussion

This study is the first to evaluate associations of multiple toxic elements with telomere length during pregnancy and in the newborn child. Importantly, the direction of the associations appeared to be exposure specific, suggesting different mechanisms. Further, the associations seemed to be tissue specific, which has previously been shown for TL in response to oxidative stress in mice [41]. More specifically, exposure to boron and antimony was associated with shorter maternal leukocyte rTL, while lithium seemed to predict for longer rTL. A doubling of the concentrations of serum boron (median 135 μg/L) and blood antimony (median 2.6 μ/L) was associated with shorter rTL by about 0.2 SD and 0.3 SD, respectively, while a similar increase in blood lithium (median 24 μg/L) was associated with an increase in rTL by almost 0.4 SD. Maternal arsenic exposure, assessed as concentrations in the blood, urine, and placenta (median 2.2 μg/L, 124 μg/L, and 8.4 μg/kg, respectively), was associated with longer telomeres in the placenta, by about 0.2 SD for each doubling of the arsenic concentration. For the newborns, the main finding was an inverse association between lead concentrations and rTL in the cord blood, and this appeared to be stronger in boys than in girls. A doubling of the cord blood lead concentrations (median 14 μg/L) corresponded to shorter rTL by 0.6 SD in the newborn boys. The clinical relevance of these associations is difficult to foresee as several factors may influence the telomere dynamics during childhood and later in life [19, 42]. More research is warranted.

There is some support in the literature for the present findings, especially concerning arsenic. Arsenic is a potent carcinogen and toxicant, and early-life exposure appears to markedly increase the disease risk later in life [43]. In the present study, arsenic concentrations in both maternal blood, urine and placenta were positively associated with placental rTL. We have previously found positive associations between arsenic exposure and telomere length in blood of adults in the same Andean area [44, 45]. Similar findings were reported for adults in Bangladesh [46] and India [47] and among adolescents in Nepal [26]. In our mother–child cohort in rural Bangladesh, the children’s urinary arsenic below 45 μg/L was positively associated with TL at 9 years of age [28]. At concentrations higher than 45 μg/L, both the prenatal and childhood arsenic exposure was inversely associated with TL. Although arsenic exposure during pregnancy has been associated with oxidative stress and inflammation in the placenta [48], we did not find any change in the association of arsenic with rTL by adjustment for the included antioxidants. The mechanism behind an arsenic-related elongation of telomeres in the placenta may rather be through stimulating the expression of TERT, the catalytic subunit of telomerase, which was found to be positively associated with arsenic exposure in adults in the same area as the present study [44]. However, what longer telomeres mean for placental function or child development is not known; indeed, the knowledge about placental rTL is sparse [5].

The neurotoxic metal lead readily passes the placenta to the fetus, and we observed an inverse association between the low-to-moderate cord blood lead concentrations (median 16 μg/L, range 6.3–60 μg/L) and cord blood rTL. Lead is a pro-oxidant [49], but the indicated association with cord blood rTL was not affected by the zinc status, which was the only other studied factor associated with cord blood rTL. The association with lead was found only for the newborn boys. Interestingly, a recent study suggested that male fetuses are particularly susceptible to maternal exposure effects on newborn TL [14], and males appear more susceptible to neurotoxicity in relation to prenatal lead exposure [50, 51]. An inverse association between blood lead and telomere length has been previously reported for 8-year-old children (*n* = 99) with low-to-moderate exposure to lead from industrial emission [27].

We found associations between maternal rTL and the exposure to lithium and boron through drinking water during pregnancy, which we previously found associated with lower size at birth [31, 32]. Since these exposures were correlated (*r*_S_ = 0.77 for serum boron and whole blood lithium), the effects should be interpreted with caution, although both associations became stronger after mutually adjusted for. We found no previous data on the impact of boron exposure on TL, but long-term medical lithium treatment in bipolar disorder, which, however, implies much higher doses than those from drinking water in the present study, has in several studies been found to be associated with markedly longer leukocyte telomeres, see, e.g., [52, 53].

Blood antimony concentrations were inversely associated with rTL in maternal leucocytes. This is in agreement with a previous study, based on the US NHANES database, which showed shorter rTL in relation to increasing urinary concentration of antimony (geometric mean 0.12 μg/L) in adults, proposed to be mediated via oxidative stress [54]. The blood antimony concentration in the present study (median 2.6 μg/L) corresponded to slightly higher urinary concentrations of antimony (median 0.18 μg/L) than in the American study, and the association seemed to weaken markedly by adjustment for zinc and folate.

In line with telomere physiology and function [3], maternal rTL decreased with increasing age, and rTL was about 30% longer in the cord blood than in the maternal blood, on average. Also, the inter-individual variation in rTL was markedly larger in the maternal blood (coefficient of variation 0.31) than in the cord blood (0.17), probably reflecting the many influential factors on the telomere dynamics during a life time. The placenta, a temporary organ, showed the shortest rTL, about 60% of that in the cord blood. In accordance with previous studies [55], placental rTL decreased with increasing gestational week at birth (about 1.5 SD over the range of 32–43 weeks in the studied women). Paternal age, known to be associated with longer telomeres in the offspring [15, 40], was associated with longer rTL in the placenta, which is largely of fetal origin, but not with cord blood rTL.

The strengths of the study include the measurement of multiple toxic exposures in different biomarker media in mother–newborn pairs, as well as rTL in the same media. Nearly all women in the study were nonsmokers with essentially no alcohol consumption, and the study area has minimal industrial or traffic pollution, factors that may affect fetal rTL [17, 56]. Other factors such as coca chewing, a common practice in the study area, was not associated with telomere length. Limitations of the study include the fairly small number of participants and the lack of cord blood and placenta samples for all women. Also, we had no useful data for initial power calculations. We measured leukocyte telomeres without adjusting for variations in leukocyte types or for placental cell type. Some of the studied elements, e.g., arsenic, boron, lithium, and cesium, were all found in varying concentrations in the drinking water in the study area and are to some extent correlated and could have led to residual confounding in some of the associations.

## Conclusions

This study adds evidence that environmental exposure to toxic metals during pregnancy may alter telomere length. We found more associations with rTL in maternal blood leukocytes during pregnancy (inverse associations with boron and antimony, positive association with lithium), than in the placenta (positive with arsenic) and cord blood (inverse with lead). Nutritional antioxidants did not generally influence the associations. More research is needed in other populations, to verify the present findings. Also the potential long-term public health impact of toxicant-related changes in rTL needs to be elucidated.

## Additional file

Additional file 1: Exploring telomere length in mother–newborn pairs in relation to nutritional factors and exposure to multiple toxic metals and potential modifying effects by nutritional factors. **Table S1**. Primers and thermocycling profiles of qPCR for telomeres and *HBB*. **Table S2**. Multivariable-adjusted linear regression between biomarkers of nutrition (antioxidants) during pregnancy, in the placenta or in the cord blood, and concurrent relative telomere length in the maternal blood, placenta, and cord blood. **Figure S1**. Scatter plots, with Lowess lines, of associations between toxic metals and rTL in maternal blood leucocytes. **Figure S2**. Scatter plots, with Lowess lines, of associations between toxic metals and rTL in the placenta. **Figure S3**. Scatter plots, with Lowess lines, of associations between toxic metals and rTL in cord blood leucocytes. (PDF 887 kb)

## Abbreviations

BMI: Body mass index
rTL: Relative telomere length
