# Supplementary material for: Exploring telomere length in mother–newborn pairs in relation to exposure to multiple toxic metals and potential modifying effects by nutritional factors
Source: BMC Med. 2019 Apr 11;17:77. doi: 10.1186/s12916-019-1309-6 (PMC6458832; doi:10.1186/s12916-019-1309-6)
Supplement: Supplementary file 1 — Exploring telomere length in mother–newborn pairs in relation to nutritional factors and exposure to multiple toxic metals and potential modifying effects by nutritional factors. Table S1. Primers and thermocycling profiles of qPCR for telomeres and HBB. Table S2. Multivariable-adjusted linear regression between biomarkers of nutrition (antioxidants) during pregnancy, in the placenta or in the cord blood, and concurrent relative telomere length in the maternal blood, placenta, and cord blood. Figure S1. Scatter plots, with Lowess lines, of associations between toxic metals and rTL in maternal blood leucocytes. Figure S2. Scatter plots, with Lowess lines, of associations between toxic metals and rTL in the placenta. Figure S3. Scatter plots, with Lowess lines, of associations between toxic metals and rTL in cord blood leucocytes. (PDF 887 kb) [file 12916_2019_1309_MOESM1_ESM.pdf]

Additional File 1 March 2019

Exploring telomere length in mother-newborn pairs in relation to nutritional factors and exposure to multiple toxic metals and potential modifying effects by nutritional factors

Maria Herlin[1], Karin Broberg[1], Annachiara Malin Igra[1], Huiqi Li[2, 3], Florencia Harari[1, 3], Marie Vahter[1]

1. Institute of Environmental Medicine, Karolinska Institutet, P.O. Box 210, SE-171 77, Stockholm, Sweden.
2. Division of Occupational and Environmental Medicine, Department of Laboratory Medicine, Lund University, Lund, Sweden.
3. Department of Occupational and Environmental Medicine, Sahlgrenska University Hospital and University of Gothenburg, Gothenburg, Sweden.

Content

Table S1. Primers and thermocycling profiles of qPCR for telomeres and *HBB*.

Table S2. Multivariable-adjusted linear regression between biomarkers of nutrition (anti-oxidants) during pregnancy, in placenta or in cord blood and concurrent relative telomere length in maternal blood, placenta and cord blood.

Figure S1. Scatter plots, with Lowess lines, of associations between toxic metals and rTL in maternal blood leucocytes.

Figure S2. Scatter plots, with Lowess lines, of associations between toxic metals and rTL in placenta.

Figure S3. Scatter plots, with Lowess lines, of associations between toxic metals and rTL in cord blood leucocytes.

Table S1. Primers and thermocycling profiles of qPCR for telomeres and *HBB*.

Primers		
Telomere	Forward	5'-CGG TTT GTT TGG GTT TGG GTT TGG GTT TGG GTT TGG GTT-3'
	Reverse	5'-GGC TTG CCT TAC CCT TAC CCT TAC CCT TAC CCT TAC CCT-3'
HBB	Forward	5'-TGT GCT GGC CCA TCA CTT TG-3'
	Reverse	5'-ACC AGC CAC CAC TTT CTG ATA GG-3'
Thermocycling profiles		
Telomere	95 °C 3 min + 25 cycles (95 °C 15 s + 56 °C 1 min)	
HBB	95 °C 3 min + 40 cycles (95 °C 3 s + 60 °C 20 s)	

Table S2. Multivariable-adjusted linear regression between biomarkers of nutrition during pregnancy, in placenta or in cord blood and concurrent relative telomere length in maternal blood, placenta and cord blood.

	Model	**Maternal blood rTL** (n=169)<br>B (95% CI)	p	**Placenta rTL** (n=99)<br>B (95% CI)	p	**Cord blood rTL** (n=88)<br>B (95% CI)	p
Zinc		Blood (mg/L)		Placenta (mg/kg)		Cord blood (mg/L)	
	1	0.046 (0.006; 0.086)	**0.025**	-0.026 (-0.062; 0.010)	0.160	-0.087 (-0.138; -0.036)	**0.001**
	2	0.058 (0.018; 0.097)	**0.004**	-0.035 (-0.070; 0.0006)	0.054	-0.070 (-0.127; -0.014)	**0.016**
	3	0.063 (0.023; 0.103)[c]	**0.002**	-0.020 (-0.056; 0.017)	0.290		
Selenium		Serum (µg/L)		Placenta (µg/kg)		Cord serum (µg/L)	
	1	-0.0027 (-0.0062; 0.0009)	0.139	-0.0005 (-0.0017; 0.0006)	0.362	0.0001 (-0.002; 0.002)	0.961
	2	-0.001 (-0.005; 0.002)	0.488	-0.0006 (-0.0018; 0.0005)	0.280	-0.001 (-0.003; 0.002)	0.524
Folate		Serum folate (nmol/L)		Serum folate (nmol/L)		Serum folate (nmol/L)	
	1	-0.011 (-0.019; -0.002)	0.018	0.001 (-0.007; 0.009)	0.733	0.003 (-0.001; 0.007)	0.195
	2	-0.012 (-0.021; -0.003)	**0.007**	0.0049 (-0.003; 0.013)	0.211	0.003 (-0.002; 0.008)	0.237
	3	-0.013 (-0.021; -0.004)	**0.005**				
Vitamin D_3		Plasma vitamin D (nmol/L)		Plasma vitamin D (nmol/L)		Plasma vitamin D (nmol/L)	
	1	0.0004 (-0.0027; 0.0034)	0.820	0.0032 (-0.0219; 0.0860)	**0.241**	0.0010 (-0.0007; 0.0027)	0.255
	2	0.0009 (-0.0022; 0.0040)	0.584	0.0039 (0.0009; 0.0069)	**0.012**	0.0008 (-0.0011; 0.0026)	0.421
	3			0.0037 (0.0007; 0.0067)	**0.017**		

Model 1. Unadjusted.

Model 2. Adjusted for mother´s age, pre-pregnancy BMI, and education; in placenta models also for gestational week at birth; in cord blood models also for gestational age at birth and birth weight.

Model3. Further adjusted for other nutrients. Maternal rTL: zinc and folate. Placenta rTL: zinc, vitamin D_3 and season.

Figure S1. Scatter plots of associations between toxic metals and rTL in maternal blood leucocytes. Lowess lines indicated.

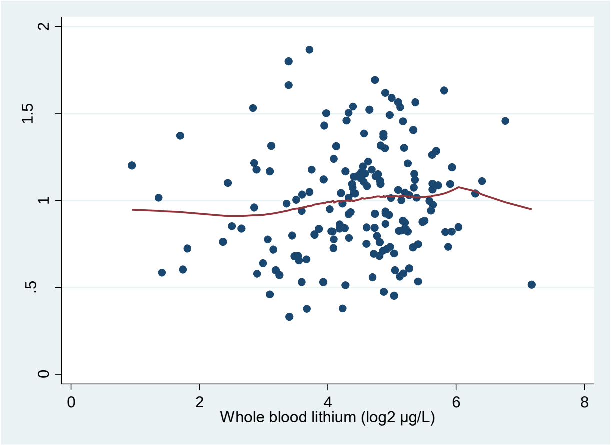

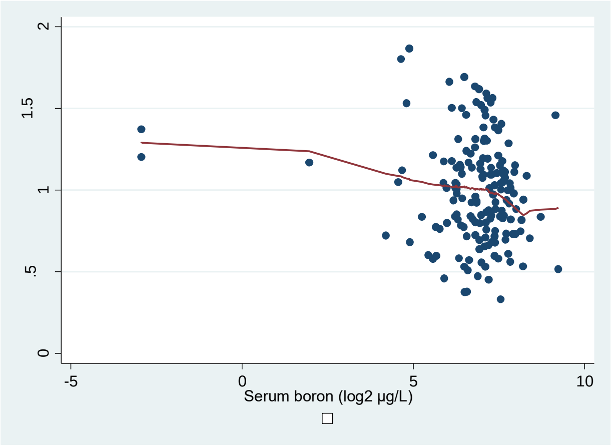

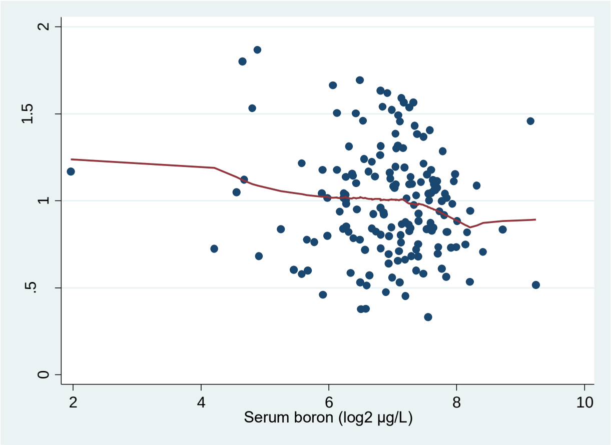

Two outliers at log2 serum boron <1 excluded

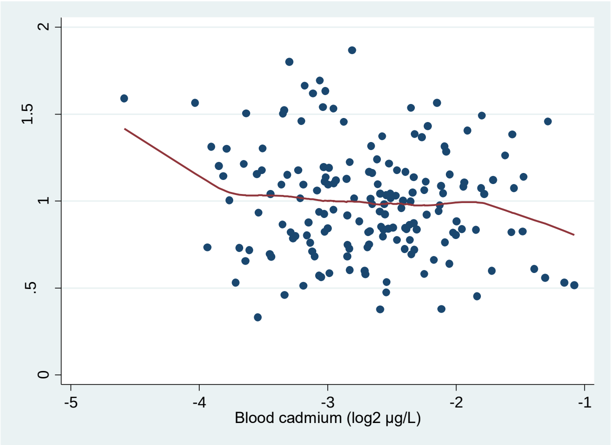

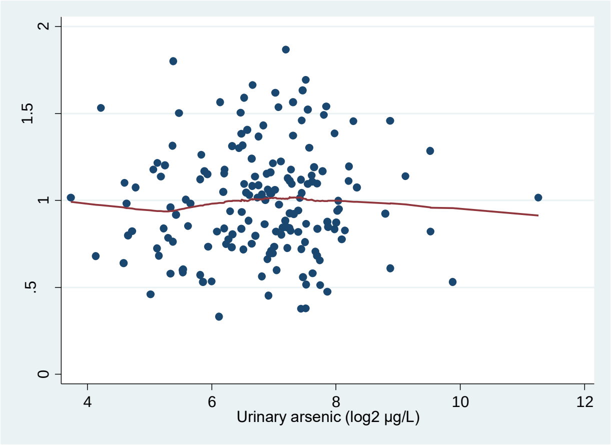

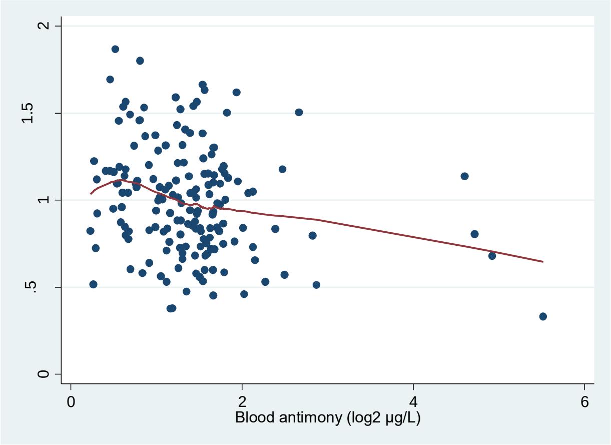

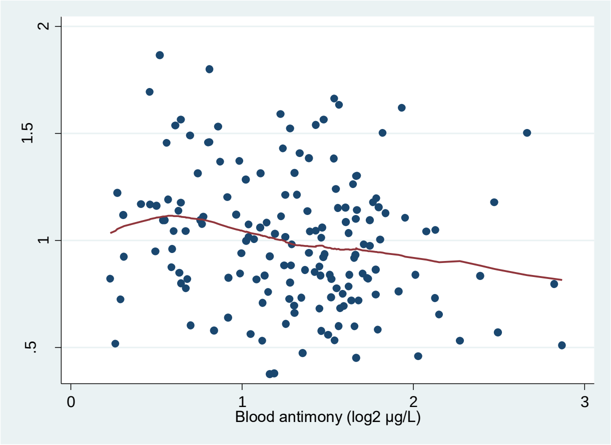

Four outliers at log2 blood antimony > 4 excluded

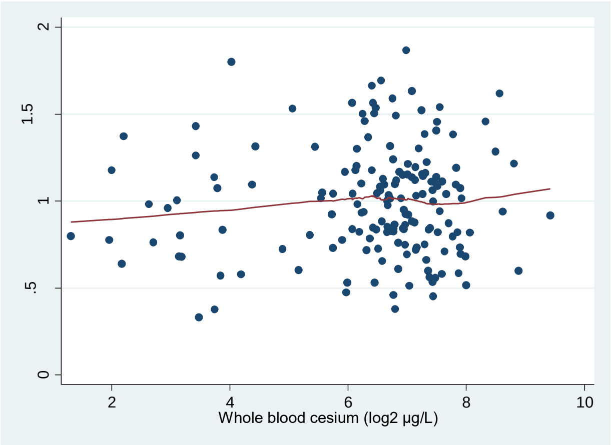

0
.5
1
1.5
2
2
4
6
8
10
Whole blood cesium (log2 µg/L)

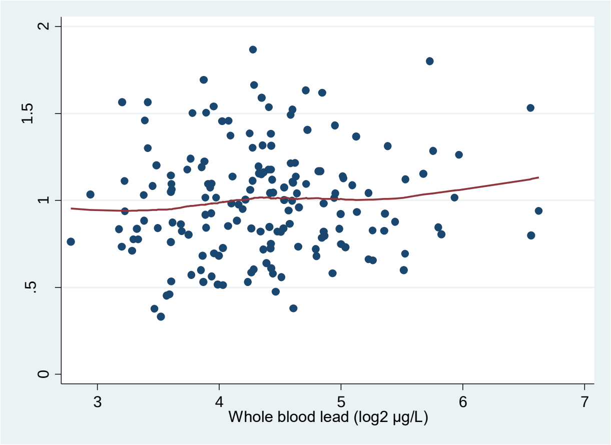

0
.5
1
1.5
2
3
4
5
6
7
Whole blood lead (log2 µg/L)

Figure S2. Scatter plots of associations between toxic metals and rTL in placenta. Lowess lines indicated.

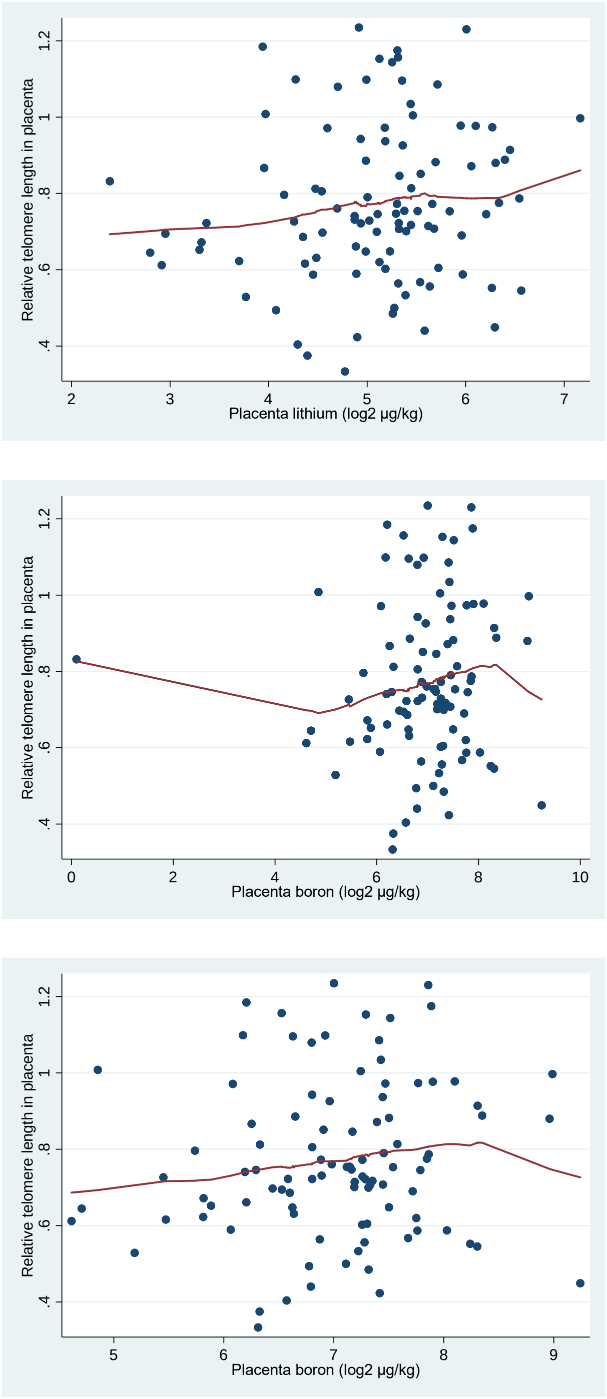

One outlier at log2 placenta boron <2 excluded

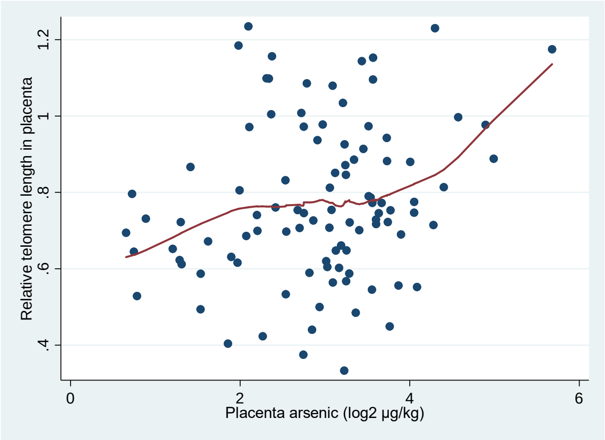
Relative telomere length in placenta
.4
.6
.8
1
1.2
0
2
4
6
Placenta arsenic (log2 µg/kg)

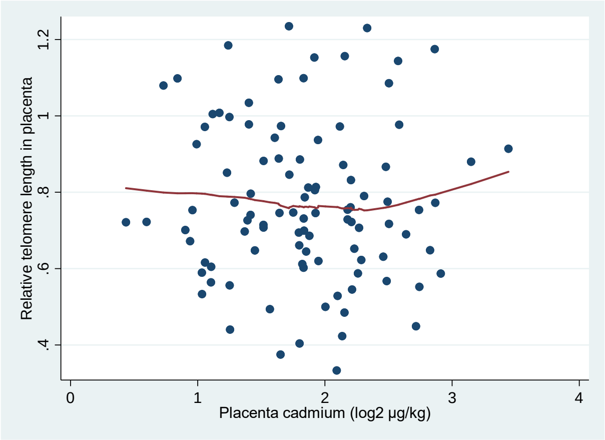
Relative telomere length in placenta
.4
.6
.8
1
1.2
0
1
2
3
4
Placenta cadmium (log2 µg/kg)

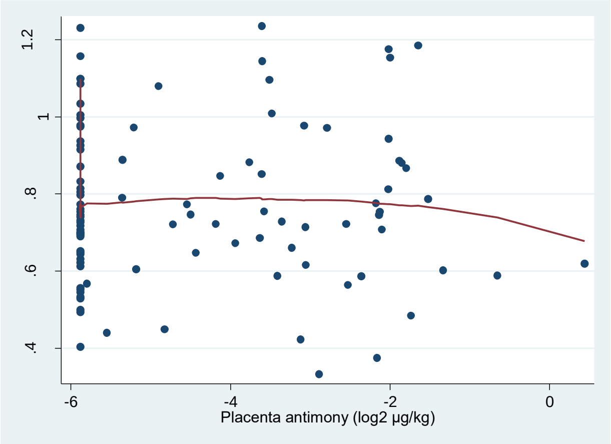
.4
.6
.8
1
1.2
-6
-4
-2
0
Placenta antimony (log2 µg/kg)

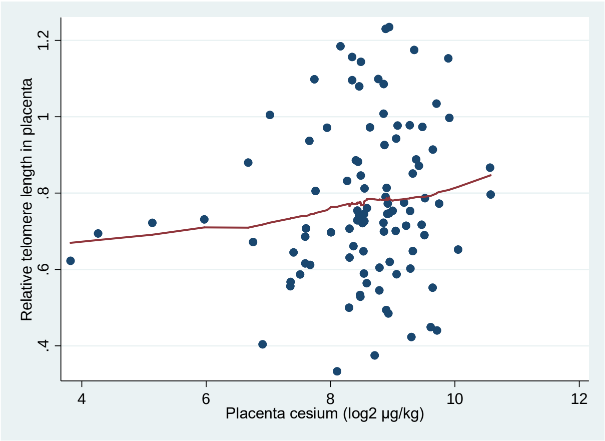
Relative telomere length in placenta
.4
.6
.8
1
1.2
4
6
8
10
12
Placenta cesium (log2 µg/kg)

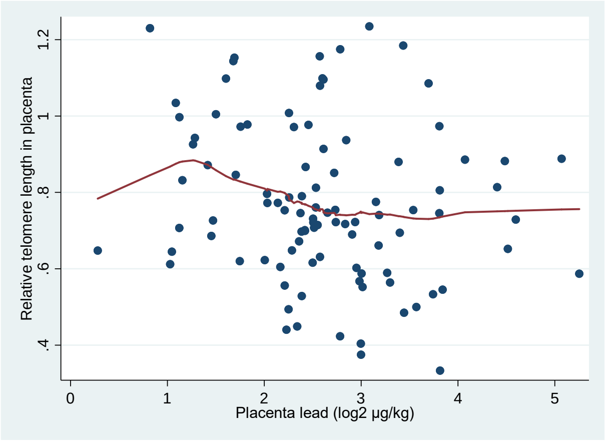

Relative telomere length in placenta
.4
.6
.8
1
1.2
0
1
2
3
4
5
Placenta lead (log2 µg/kg)

Figure S3. Scatter plots of associations between toxic metals and rTL in cord blood leucocytes. Lowess lines indicated.

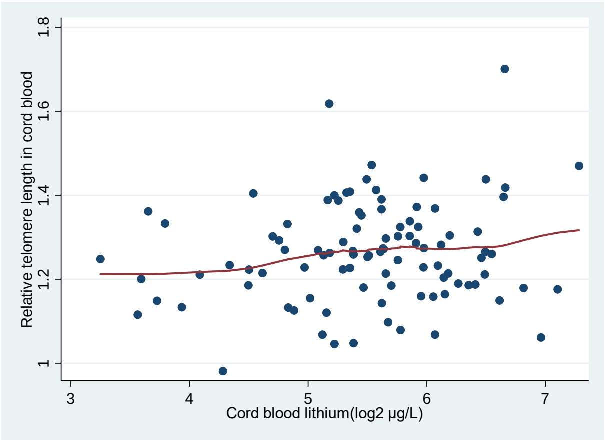

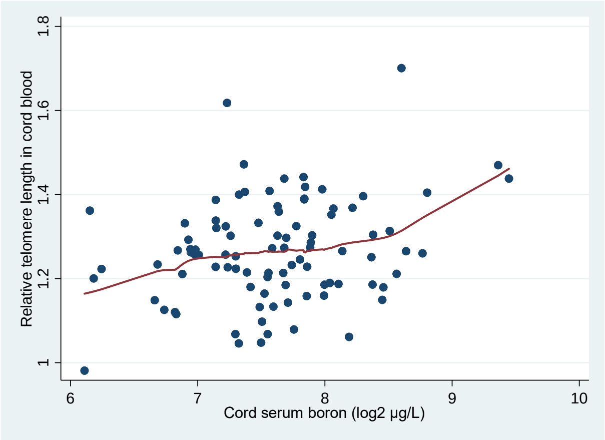

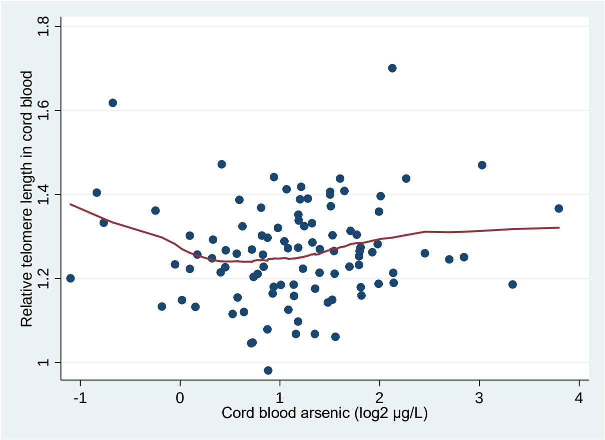

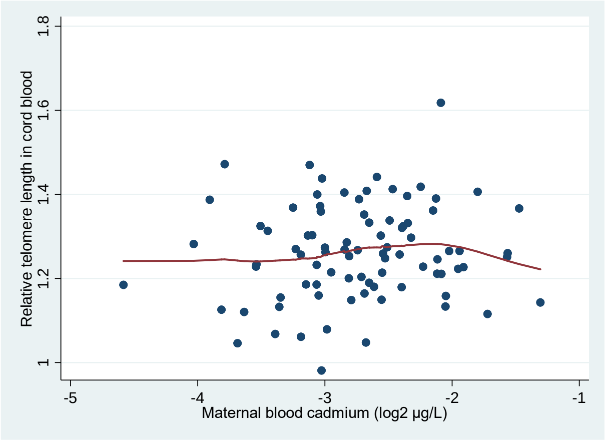

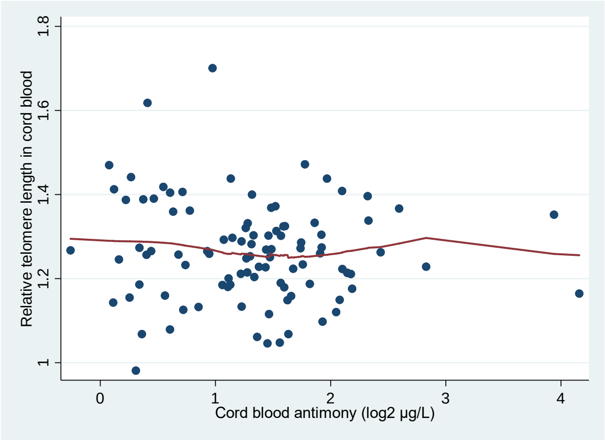
Relative telomere length in cord blood
1
1.2
1.4
1.6
1.8
0
1
2
3
4
Cord blood antimony (log2 µg/L)

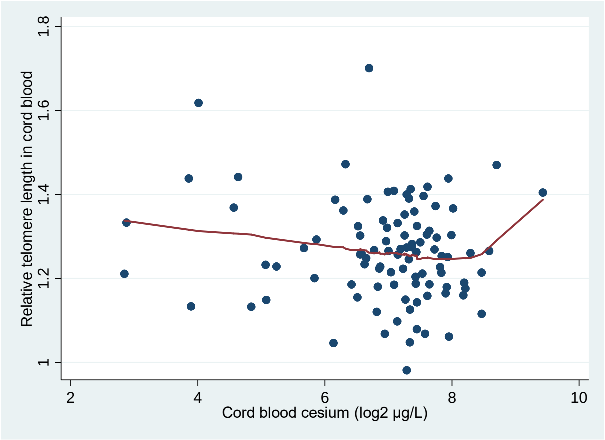
Relative telomere length in cord blood
1
1.2
1.4
1.6
1.8
2
4
6
8
10
Cord blood cesium (log2 µg/L)

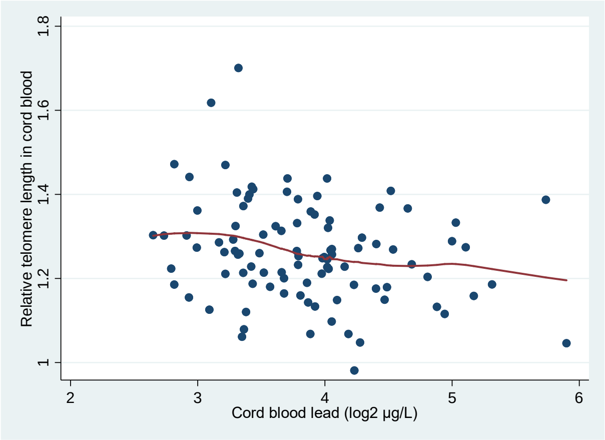
Relative telomere length in cord blood
1
1.2
1.4
1.6
1.8
2
3
4
5
6
Cord blood lead (log2 µg/L)

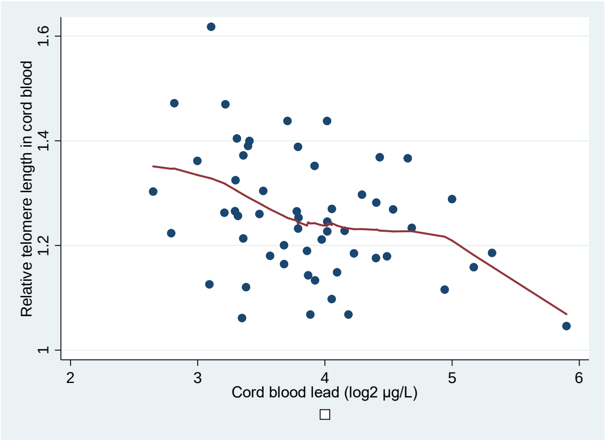

Scatter plot for cord blood lead and rTL in BOYS.

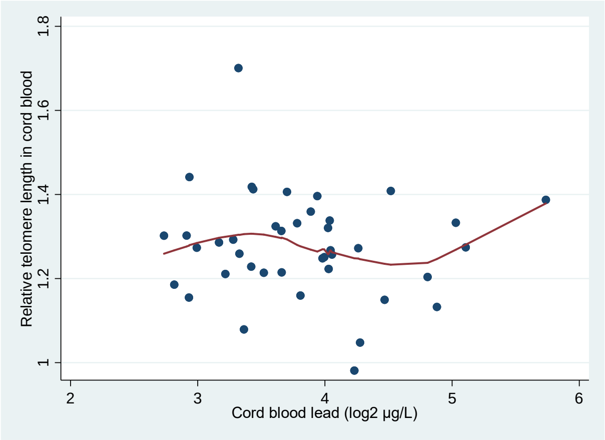

Scatter plot for cord blood lead and rTL in GIRLS.
